# Review of Recent Medicinal Applications of Rhenium(I) Tricarbonyl Complexes

**DOI:** 10.3390/ijms26147005

**Published:** 2025-07-21

**Authors:** Erick Kipngetich Towett, Vuyelwa J. Tembu, Douglas Kemboi, Moses K. Langat, Amanda-Lee E. Manicum

**Affiliations:** 1 Department of Chemistry, Tshwane University of Technology, P.O. Box X680, Pretoria 0001, South Africa; towetterick@gmail.com (E.K.T.); tembuvj@tut.ac.za (V.J.T.); 2 Department of Physical and Biological Sciences, University of Kabianga, Kericho P.O. Box 2030, Kenya; dkemboi@kabianga.ac.ke; 3 Royal Botanic Gardens Kew, Richmond, Surrey TW9 3AE, UK; m.langat@kew.org

**Keywords:** antichagasic, antimalarial, antimicrobial, diagnostics, Re(I) tricarbonyl, therapeutics

## Abstract

The use of metal-based complexes is currently taking centre stage in the field of nanomedicine for the treatment and control of various ailments. Rhenium(I) tricarbonyl complexes have frequently been evaluated in vitro for their anticancer activities, and a few have advanced to in vivo and clinical trials, owing to the distinct application characteristics of these complexes. Their inception in drug development is key. This study explores a detailed chronological overview of the medical applications of Re(I) tricarbonyl complexes over the past six years (2019–2024), focusing on their applications and clinical tests in the control and management of various ailments. An in-depth examination of their activities in anticancer treatments, Chagas disease, antifungal infections, antimalarial, and microbial infections was conducted, comparing the complexes to various standard antibiotics, conventional antimalarial drugs, antifungals, and standard anticancer agents.

## 1. Introduction

The field of medicine and the healthcare system is a progressive unit with immense pressure on pharmaceutical industries and academic research institutions to conduct research towards developing drugs that are effective and specific in action [1]. The emergence of novel infectious diseases, as well as the re-emergence of other diseases due to resistance, puts more emphasis on the discovery of new drugs [2]. Computational chemistry and biology play a central role in drug development. The process involves a multiparameter optimisation and a target-specific drug formulation, a procedure that has shortened the development period of drugs [1].

The introduction of metal-based drugs has sought to improve the efficiency and specificity of the drugs by introducing a metal complex to enhance stability and create more reactive sites within the drugs [3,4]. This nanotechnology uses nanomaterials such as Manganese Mn(II), Mn(I), platinum (Pt), and rhenium (Re(I), (II)) [3].

The economic feasibility of Rhenium has made it a centre of concern in evaluating its medical applications. Much of the Rhenium with varied oxidation states and multi-metallic complexes have been evaluated, with little attention given to Re(I) Tricarbonyl complexes. These complexes have been observed to possess palpable antibacterial, antifungal, antimalarial, antichagasic, and anticancer activities. A comprehensive data review on these biological applications is necessary.

**Figure 1:**
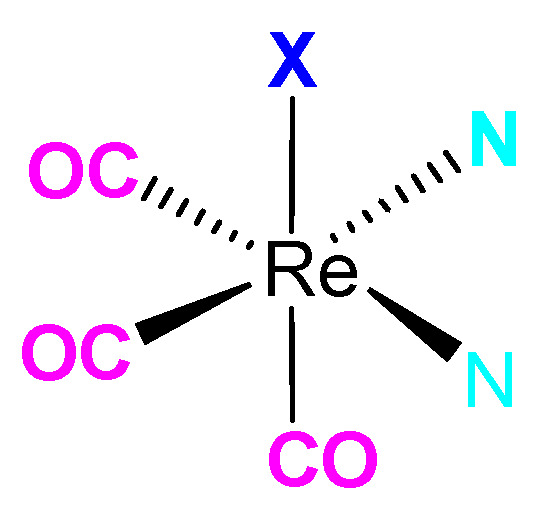
An analogous structure of [Re(I)(CO)_3_]^+^ Complex (X, *N,N’* = represents the coordinating ligands).

Re(I) Tricarbonyl complexes contain a low-spin d^6^ electronic configuration as well as stability of CO ligands, making it probable for substitution of the ligands in the [Re(I)(CO)_3_]^+^ core (Figure 1), hence its usefulness in radiopharmaceutical medicine. The metal neutrality in the Re(I) complex makes it a more advantageous property as opposed to its radioisotope state. The complex also has a relatively small size as compared to its stability and inertness, serving as a probable benefit in nanomedicine applications [5,6].

The luminescent cum phosphorescent properties of the [Re(CO)_3_]^+^ complexes give it a characteristic application as a photosensitiser and as a bio-imaging agent. The remarkable C-O stretching frequency enables many of the rhenium complexes for imaging [7].

**Figure 2:**
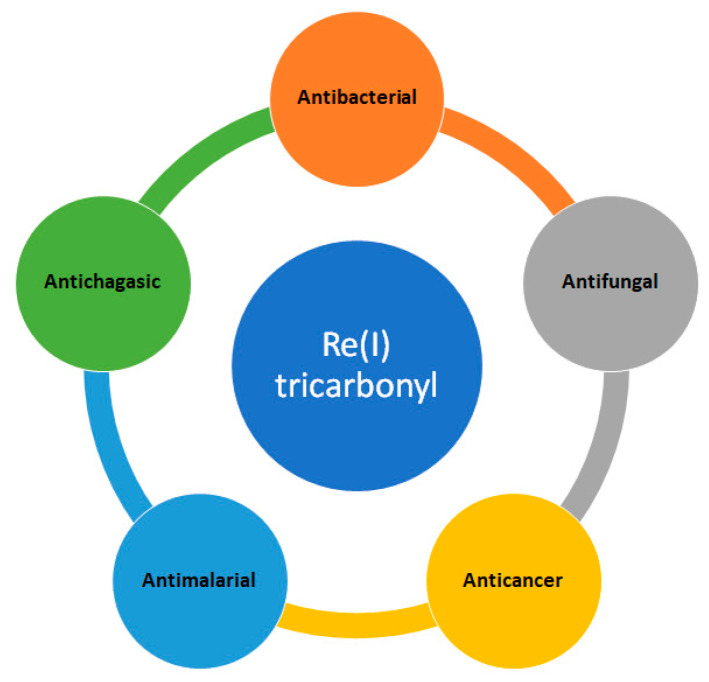
Medical applications of Re(I) tricarbonyl complexes.

These structural and chemical characteristics of Re(I) tricarbonyl complexes make them useful in the management of varied medical conditions, as shown in Figure 2.

Re(CO)_3_-*N,N’*-diamine derivatives have been evaluated as photocatalysts in CO_2_ reduction as well as in photodynamic therapy (PDT) (**1**–**3**) (Table 1). These complexes have also shown a desirable antibacterial activity against *Streptococcus aureus* and *E. coli*, with complex **3** showing the lowest MIC value of 50 µg/mL [8].

The introduction of bis-quinoline to the *N,N’*-diamine complexes enhances their antibacterial activities, wherein complex **9** exhibited notable antibacterial activity against *S. aureus* (MIC = 0.0002 mM) and *E. coli* (MIC = 0.023 mM) [8]. The activity of the complex did not change when the methicillin-resistant strain of *S. aureus*, as well as the colistin-resistant strain of *E. coli*, were used (Table 2), an indication that the complex is a prospective candidate in drug development [8,9,10].

Most of the complexes herein reported have shown poor antifungal activities against *Candida albicans*, with only complex **12** showing a potent activity of 0.00062 mg/mL against *C. albicans* (Table 3) [15,16]. It has been reported that both antifungal and antibacterial agents work by generating singlet oxygen responsible for enhancing drug penetration to the pathogenic organs, hence inhibiting their growth [8,16].

The derivatisation of triazoles with [Re(CO)_3_]^+^ enhances the antimalarial activities of complexes. Complex **14** of the azole group of compounds shows an interesting IC_50_ value of 4.16 µM against *P. falciparum (*Table 4) [18,19]. Conversely, [Re(CO)_3_]^+^ of clotrimazole derivatives show desirable antichagasic activities against *T. cruzi*, with complex **18** showing the least IC_50_ value of 3.48 ± 0.98 µM against *T. cruzi epimastigotes* (Table 5) [20,21,22]. However, many of the complexes have been dismally evaluated for their antichagasic activities, creating a gap that should be filled through rigorous research and development.

Several complexes have been evaluated for anticancer activities against different cancer cell lines, with the complexes ranging from simple *N,N’*-diamine (**23**–**28**), sulfonated [Re(CO)_3_]^+^ complexes (**29**), benzo thiols derivatives (**30**–**33**), 1,10-phenanthroline derivatives (**36**–**41**), [Re(CO)_3_]^+^ acetylacetone tricarbonyl-1,2-dimethylimidazole (**43**) to the Re(I) binuclear carboline, and 1,10-phenanthroline derivates (**44**–**48**) [6,23,24,25,26,27,28,29,30] in each case, the anticancer activities of these complexes were compared with those of cisplatin (**42**), the positive control, as shown in Table 6. However, none of the rhenium complexes have passed through clinical trials. There has also been a series of toxicities associated with the complexes, which in turn have limited their applications. The present review seeks to evaluate the medicinal applications of Rhenium(I) complexes with a narrower approach to the antimicrobial, antifungal, antimalarial, antichagasic, and anticancer activities of [Re(CO)_3_]^+^ complexes published over the past six years (2019–2024). The data herein were extensively analysed from scientific databases such as Google Scholar, Scopus, PubChem, Sci-Finder, and Science Direct.

## 2. Medical Applications of Re(I)Tricarbonyl Complexes

The Re(I) tricarbonyl core has recently emerged as an ideal metal synthon in medicinal applications due to the biocompatibility, low toxicity, and unique electronic properties that the complexes of this metal possess [6]. The applications that make these [Re(CO)_3_]^+^ complexes valuable in medicine are diagnostic imaging, cancer treatment, and antibacterial capabilities. This is due to the versatility of the metal core, where scientists can modify the ligands coordinated in the octahedral sphere, allowing for various fine-tuning to optimise the complex properties [6,34]. In addition, the [Re(CO)_3_]^+^ complexes with diamine (*N,N’*) coordinated ligands are the desired complexes owing to their desirable luminescent properties. They are simply synthesised at room temperature and can easily emit light in the UV–VIS region; their characteristics, however, are dependent on the conditions set and the type of ligands used. Due to these remarkable advantages, they have been formerly used as photocatalysts in the reduction of CO_2_, as sensors for different microenvironments, and as singlet oxygen sensitisers for photodynamic therapy (PDT). They have a general formula of [Re(CO)_3_(*N,N’*)X]^+^, where X represents a halide that is characteristic of yielding a triplet metal–ligand charge transfer (**^3^**MLCT).

The properties of the Re(I) complexes depend on the ligands, the solvent used, and other physical properties applied, hence yielding varied types of complexes with distinguished biomedical applications [6]. This review, therefore, seeks to identify these complexes and their biomedical applications for the past six years (2019–2024), with an in-depth look into some of their antimicrobial, antifungal, antimalarial, antichagasic, and anticancer activities.

### 2.1. Antibacterial Applications

The World Health Organisation (WHO) terms antimicrobial resistance (AMR) as a global challenge affecting countries at all levels [35]. It is the leading cause of antibiotics’ ineffectiveness worldwide. The spread of drug-resistant pathogens is occurring at an alarming rate, as clinical innovation in antibiotics is drying up, posing a challenge to the growing antimicrobial resistance (AMR) menace. The increasing antimicrobial resistance (AMR) worldwide has strained the economic power and health systems of many countries, a concern that demands urgent attention from all sectors [35]. However, the recent increasing adoption of metal-based complexes has shown a green light to end the AMR menace, even though a few of them have undergone clinical trials.

Irradiating complexes **1**, **2**, and **3** with UV–Visible light at 365 nm gives an advanced antibacterial activity against human pathogens *S. aureus* and *E. coli* (Table 1). Acosta et al. (2021) [8] reported significant activities of irradiated complexes against selected human pathogens compared to non-irradiated ones. The irradiated form of complex **1** showed a profound activity against both *S. aureus* and *E. coli*, with an MIC of 50 µgmL^−1^. Conversely, the irradiated form of complex **2** provoked cell arrests against the Gram-negative bacterial strain *E. coli* at 300 µgmL^−1^, whereas complex **3** had a significant activity against *S. aureus* at 50 µgmL^−1^.

Sovari et al. (2020) [24] evaluated the antimicrobial potency of complexes (**4**–**8**) against Gram-positive bacterium *S. aureus* and its methicillin-resistant strain (MRSA), *Enterococcus faecium*, and *Listeria monocytogenes* and Gram-negative bacterial strain *Pseudomonas aeruginosa*, wherein complexes **4**–**8** showed profound activities against *S. aureus* NCTC 6171 and *S. aureus* ATCC 43300 (MRSA) with minute concentrations of less than 0.8 µM (700–800 ng/mL) (Table 2) [24].

The complexes **4**–**8** demonstrated potent activity, approximately eight times greater than that of linezolid [MIC = 5.9 µM], indicating that the complexes perform significantly better than the approved antibiotics in the treatment of methicillin-resistant *S. aureus* [24].

In vivo, investigations of these complexes were evaluated in the zebrafish model [36]. The results disclosed that rhenium complexes with anti-staphylococcal activity were of low toxicity at higher dosage as compared to corresponding MICs against MRSA and *S. aureus* NCTC 6571, and thus can serve as a probable lead in the control and treatment of bacterial infections [8].

*P-N* bidentate (**1**–**3**), as demonstrated by Acosta et al. (2021) [8], showed promising activities against some of the selected human pathogens *S. aureus* and *E. coli*, with the lowest MIC value of 50 µgmL^−1^. However, Sovari et al. (2021) [16] demonstrated that the introduction of an electron donor to the ligand, as well as increasing the length of the side chain, will enhance the antibacterial activities of the complexes, which was then affirmed via zebrafish tests by Briggs (2002) [36].

On the other hand, as demonstrated by Frei et al. (2020) [13], the introduction of a bis-quinoline ligand to the Re(CO)_3_ core increases its antibacterial activities. The complexes (**9**–**11**) are the derivatives of bis-quinolone, which were subjected to an antibacterial activity test against Gram-positive bacterium *S. aureus* and Gram-negative bacterium *E. coli*, as well as their resistant species MRSA and colistin-resistant *E. coli* strains, wherein their MIC values (mM) are tabulated in Table 2.

Complex **9** showed profound antibacterial activity against *S. aureus* and *E. coli*, with corresponding MIC values of 0.0002 and 0.023 mM, respectively. It was also noticed that these antibacterial activities did not change in any way when Methicillin-resistant *S. aureus* (MRSA) and colistin-resistant *E. coli* strains were used [13]. This is evident that the complex has potent antibacterial activities against both Gram-negative and Gram-positive bacterial strains, hence a good candidate for the development of antibiotics [13,37]. Similar antibacterial activities were observed by Romão et al. (2024) [38], where pyridylbenzimidazole derivatives (**12**, **13**) showed interesting activities against *S. aureus.* The advanced activities are associated with the presence of phenylic ring in the ligands.

As reported by Frei et al. (2020) [13], irradiating the complexes at around 365 nm enhances their activities. This is because it aids in the release of singlet oxygen, which enhances penetration of antibiotics into the bacterial membrane; this was also evaluated and affirmed by Acosta et al. (2021) [8], where a twofold increase in the antibacterial activities of complexes **1**–**3** were reported after a 365 nm irradiation.

Meagre antibacterial activities of Re(CO)_3_ complexes against Gram-negative bacterial strains have been reported in several studies. Frei et al., (2020) [13], Betts et al., (2020) [37], and Sovari et al., (2021) [16] affirm this when poor antibacterial activities of complexes **1**–**11** against Gram-negative bacterium *E. coli* were reported (Table 1 and Table 2). This may be attributed to the resistance of the bacterium, aided by a thick bacterial membrane as well as the presence of an efflux pump, which helps in evacuating antibiotic accumulation within their systems [15].

From 2019–2024, many Re(I) complexes have been evaluated for their antibacterial activities against varied bacterial strains; however, a few have been tested in varied animal models to establish their safety for human uptake, as compared to their anticancer activities [11,16]. Many of the studies have largely focused on sensitivity tests on *S. aureus* and *E. coli*, with no concern for some neglected tropical pathogenic bacterial strains, such as *Mycobacterium ulcerans* which causes *Buruli* ulcers, a disease characterised by the formation of large ulcers on the skin and reported to cause acute paralysis in humans, and consequently, may cause skin cancer if not treated early [39]. Secondly, *Gardnerella vaginalis* is a common bacterial infection that causes vaginal microbiota imbalance; the condition, however, is not life-threatening, but an untreated condition that may cause serious complications during pregnancy and acts as a route for other sexually transmitted infections [40]. The two bacterial infections are among many other neglected pathogens that silently cause death; hence, a research call ought to be created to look into the potential of MNPs in the development of other classes of antibacterial drugs.

### 2.2. Antifungal Activities

There are currently fewer antifungal drugs in clinical trials; however, new fungal strains resistant to most current antifungals are spreading quickly around the world. Fungal infections present a dreadful trend in human health. *Candida* spp. is among this group of fungal infections, presenting the third leading cause of bloodstream infections worldwide, with a projected annual death rate of 700 annually [41,42]. For the past three decades, only three classes of antifungals have been developed and have undergone clinical practice.

**Scheme 1:**
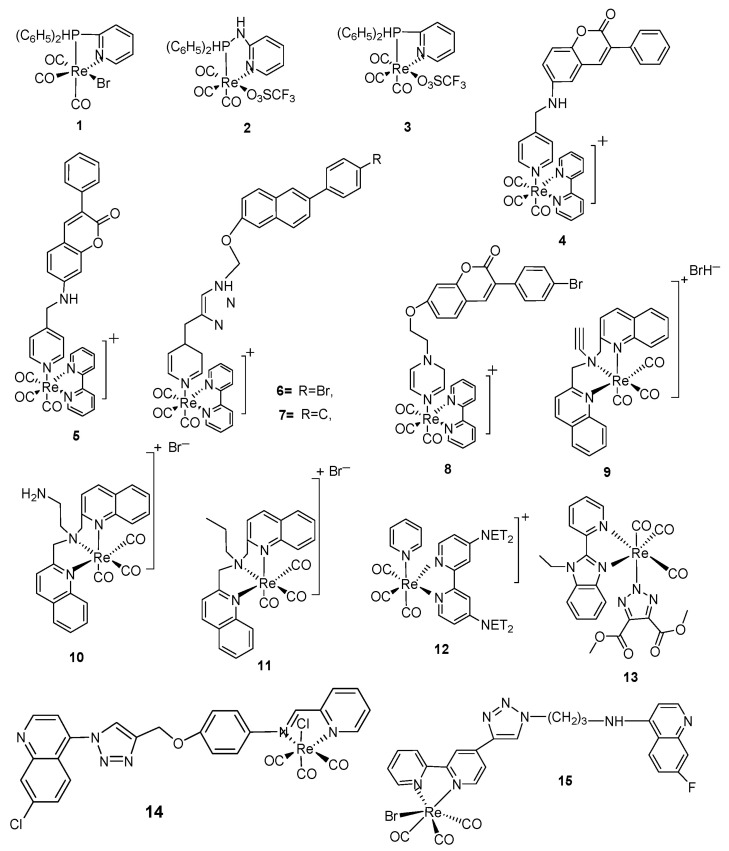

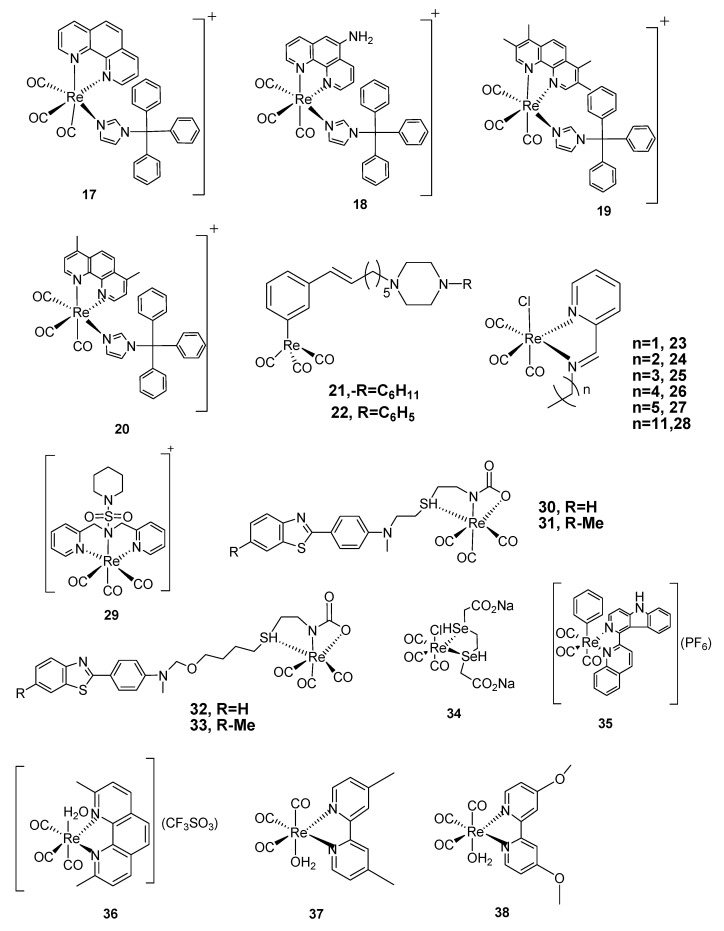

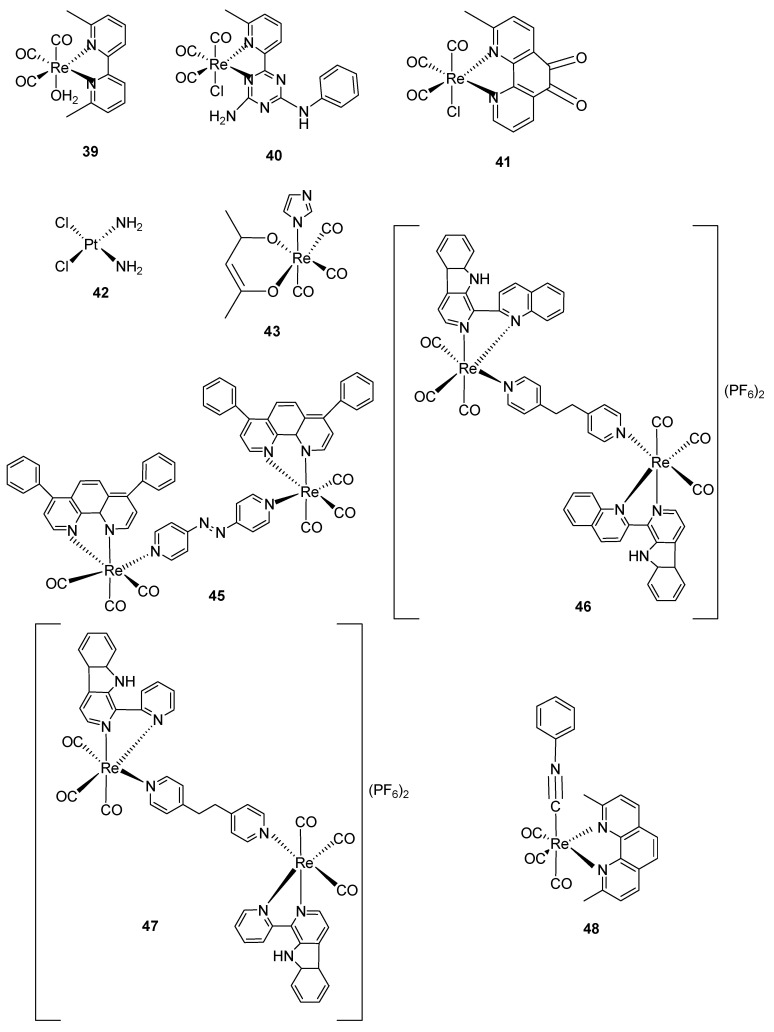
Structures of Re(CO)_3_-diphenylphosphinebromide [Re(CO)_3_PNBr] (**1**), Re(CO)_3_diphenylphosphine-iminebromide, [Re-PNNBr] (**2**), Re(CO)_3_ diphenylphosphinetriflate [RePNTfO] (**3**), 3-arylcumourin derivatives of [Re(CO)_3_]^+^ complexes (**4**–**8**), benzo-quinoline derivatives of [Re(CO)_3_]^+^ complexes (**9**–**11**) and triazoto [Re(CO)_3_]^+^ pyridylbenzimidazole derivatives (**12**, **13**), Re(CO)_3_-quinine triazole complexes (**14**–**16**), Re(CO)_3_-clotrimazole-1,10-phenanthroline (**17**), Re(CO)_3_-clotrimazole-5-amino-1,10-phenanthroline (**18**), Re(CO)_3_-clotrimazole-3,4,7,8-tetramethyl-1,10 phenanthroline- (19) and Re(CO)_3_-clotrimazole-4,7-dimethyl-1,10-phenanthroline- (20), Re(CO)_3_-nitro sulphonated complex (**29**), Re(CO)_3_-benzothiols derivatives (**30**–**33**), Re(CO)_3_-chloro- (1,4-sodium ethanoate)-acetly (**34**), Re(CO)_3_-phenyl-1,10-phenanthroline-4,4′-azopyridine (**35**), Re(CO)_3_-1,10-phenthroline derivatives (**36–41**), Cisplatin positive control (**42**), Re(CO)_3_-acetylacetone-1,2-dimethylImidazole (**43**), Re(CO)_3_-(2,9-dimethyl-1,10-phenathroline (**44**), Re_2_(CO)_6_(diphenylphenanthroline)-2-4,7-diphenyl-1,10-phenanthroline-4,4′-azopyridine (**45**), Re_2_(CO)_6_-complexes of carboline derivatives (**46**, **47**), Re(CO)_3_-2,9-dimethyl-1,10-phenanthroline-para tolylisonitrile (**48**).

The azoles, polyenes, and echinocandins are among these antifungal drugs [43]. A clear suggestion that attention is required to ensure the development of more potent antifungals. Conversely, the currently used antifungal agents have been faced with severe multidrug resistance and a synergistic cohabitation with other microbes such as *S. aureus*, causing serious polymicrobial infections to humans [42]. To prevent a second resistance crisis, new antifungal drug classes are urgently required. Metal complexes have proven to be promising candidates for novel antibiotics. However, few of these complexes have been explored for their potential application as antifungal agents, shown in Table 3 and Scheme 1.

Many of the complexes showed mild activities against the varied species of *Candida* as reported by Sovari et al. (2021) [16]. However, complex **12** showed potent antifungal activity against *C. albicans* with its corresponding MIC value of 0.00062 mg/mL, a promising activity for the development of antifungal drugs. Mansour [17], synthesised complexes of azide analogous with coordination ligands of pyridyl benzimidazole **13** and reported promising antifungal activities against the invasive species of *C. albicans* and *C. neoformans* with an MIC value of 0.032 mg/mL. These antifungal activities have no significant difference as compared to antifungal reference drugs: fluconazole, vancomycin, and colistin, an indication that their activities are desirable in the development of antifungal drugs.

As compared to antibacterial complexes, antifungal agents work by generating singlet oxygen, which is responsible for enhancing the penetration of antibiotics via the cell membrane to the organ system, hence prohibiting its multiplication [8,16]. Most of the antifungal agents, however, have not been evaluated in vivo, barring their clinical applications in drug development.

Between 2019 and 2024, a few investigations have been carried out to evaluate the antifungal potential of MNPs in the development of probable antifungal drugs, with a narrower approach to the *Candida* species only. This has left aside other life-threatening fungal strains such as *Blastomyces dermatitidis*, which causes blastomycosis disease, *Coccidioides immitis* and *Coccidioides posadasii*, which cause coccidioidomycosis diseases, and *Paracoccidioides brasiliensis*, which causes *paracoccidioidomycosis* disease. These neglected fungal pathogenic strains are prevalent in specific areas and can cause a series of respiratory and systemic infections. Secondly, no Re(I) tricarbonyl complex with antifungal activities has been evaluated in vivo, barring their clinical applications in drug development. These conditions, therefore, call for a better approach to developing metal-based antifungal agents to enhance effective treatment of varied fungal infections.

### 2.3. Antimalarial Activities

Malaria is still a public health concern, owing to its prevalence and significant impact on the health systems, especially in countries in sub-Saharan Africa [44]. In 2023, UNICEF reported about 222 million cases as of 2022, an indication that the prevalence is increasing yearly [45]. Multidrug resistance of *Plasmodium falciparum* is the main concern in the management and control of malaria. However, the introduction of metal-based drugs has been reported to scale down such resistance. The Re(CO)_3_ complexes of the quinoline triazoles ligand scaffold work best by inhibiting haemoglobin oxidation, which is key in the treatment of the most dreadful cerebral malaria [19].

Derivatising quinoline triazoles with the [Re(CO)_3_]^+^ core led to the development of more probable antimalarial complexes with profound activities [19]. The study carried out by Ishmail [19] found that complex **14**, [IC_50_ = 4.61 µM], was active against *P. falciparum* and its resistant isolate. Conversely, improved antimalarial activities were reported with complexes **15** and **16** against *P. falciparum*, and its resistant isolate with corresponding IC_50_ values of 0.356 and 0.441 µM, respectively (Table 4) [18].

The potent activity of these complexes is enhanced by the incorporation of CO, which has the characteristic advantage of preventing the polymerisation of heme, hence preventing plasmodium infections [19]. Silicon hemozoin docking reveals that the complexes work by disrupting plasmodial heme detoxification pathways, a process that aids in inhibiting the pathogenic effects of *P. falciparum*. The quinoline functionalised complexes have recorded the lowest IC_50_ value of 0.098 ± 0.008 µM against *P. falciparum* in vitro [18,19], an indication that these can be utilised in antimalarial drug development.

Over half a decade ago, many studies on the antimalarial potential of Re(CO)_3_ complexes were reported. However, a few of these complexes have been scaled up for in vivo studies and lately on clinical trials, but none have been approved for pharmaceutical applications. Secondly, more research is needed to understand the mode of action of such MNPs to improve their novel properties towards the development of viable drugs. This, therefore, calls for multi-disciplinary research and development to bring in novel metal-based antimalarials.

### 2.4. Antichagasic Activities

The treatment of CD has, over time, been hampered by the resistance of the main causative agent *T. cruzi*, and severe toxicity of the drugs [46,47]. There has been no drug development along the antichagasic line, with Benznidazole and nifurtimox, which were developed about 60 years ago, being actively used to date [20,21]. There is an urgent need, therefore, to improve the efficiency of CD treatment. The emergence of metal complexes created a new sphere for the development of varied drugs. Soba et al. (2023) [20] synthesized and characterised the multi-functionalised Re(I) (CO)_3_ complexes with varied N- bioactive monodentate clotrimazole and bidentate polypyridyl *N*,*N’* derivative of 1,10- phenanthroline ligands (Scheme 1), and four active complexes of Re(I) were synthesised: **17**, **18**, **19**, and **20**, where in each case, Hexafluorophosphate (PF6) was used as an anionic stabilizer.

Complex **19** showed the highest activity against the epimastigotes of *T. cruzi*, with its corresponding IC_50_ value of 3.48 ± 0.98 µM [20]; however, complex **17** showed the lowest selectivity index against epimastigotes of *T. cruzi*, corresponding to 0.34, an indication that the complex could be the preferred candidate for the development of antichagasic metal-based drugs (Table 5). These results on the antichagasic activities of the azole complexes of [Re(CO)_3_]^+^ conform with those of Gonçalves et al. (2024) [22], wherein the metallomics study conducted on microwave plasma spectrometry (MP-AES) reported similar IC_50_ values of these complexes.

In each case, the complexes work on inhibiting the biosynthesis of ergosterol as well as disrupting their DNA mark-ups of the epimastigotes via the use of free electrons on the *N,N’* moiety [20,21,22].

The synthesis of complexes **17**–**20**, to the best of our knowledge, are the new [Re(CO)_3_]^+^ complexes with micromolecular IC_50_ values against *T. cruzi epimastigotes* and are prospective agents in the development of antichagasic drugs. Antichagasic properties of metal complexes have not been exhaustively investigated; this, therefore, opens up another spectrum for further investigation and research along the *Trypanosoma* line. However, such complexes with the azole functional unit have been formally investigated for their antimalarial and antifungal activities as reported by Sovari et al. (2021) [16] and Ishmail (2019) [19].

Between 2019 and 2024, a few [Re(CO)_3_]^+^ derivatives have been evaluated in vivo for their antichagasic activities, and none have passed through clinical trials in preparation for metal-based drug development, an indication that the disease is a neglected tropical disease, despite causing many deaths in Latin America. There has also been limited research alongside the development of antichagasic conventional drugs, with only benznidazole and Nifurtimox being in use for the past 60 years. The two drugs have been reported to cause severe toxicity in patients, alongside them becoming less effective due to the resistance of *T. cruzi* towards their usage. This is a clear indication that a lot must be done to enhance the availability of antichagasic metal-based drugs.

### 2.5. Anticancer Activities

In recent years, organometallic compounds have emerged as promising candidates for anticancer drugs. While radioactive 186/188Re compounds are already used in cancer treatment, cold Re organometallic compounds have been studied primarily as luminescent probes for cell imaging and photosensitisers in photocatalysis. However, a growing number of studies have shown that Re organometallic complexes have anticancer properties. Several compounds have demonstrated cytotoxicity equal to or greater than that of the well-known anticancer drug cisplatin. For example, Schindler et al. (2023) [28] examined the anticancer activity of some *N,N’*-diamine complexes and their derivatives against selected cancer cell lines. Scheme 1 herein gives some of these complexes (**21** and **22**) with activities and selectivity slightly higher than those of cisplatin against colon cancer, with corresponding IC_50_ values of 18.11 µM and 22.23 µM in HT-29 and PT-45 cell lines, respectively. The cytotoxicity of complexes **21** and **22** is dependent on the size of the side chain alkyl substituent, i.e., the larger the alkyl length, the more active the complex is.

Konkankit et al. (2019) [48] further focused on the synthesis of Re(I) complexes bearing different lengths of alkyl chains side substituents (Scheme 1), where complexes **23**–**27** exhibited activity against HeLa cells with IC_50_ values going below 15 µM, which reduces with an increasing alkyl chain substituent. Complex **33**, therefore, had the highest activity as compared to the rest of the complexes, which is attributed to the increasing lipophilicity of the complexes, enhancing anticancer activities on the cancer cell lines.

Sulphonating of [Re(CO)_3_]^+^ complexes resulted in the formation of Nitro-sulphated complexes, which are formed by the addition of ligand (N (SO_2_PiP) dPa) into the *N,N’* in diamine (Scheme 1). The study revealed that the ligand part of complex **29** had more activity as compared to its complexes with corresponding IC_50_ values of 139 and 360 µM against MCF-7 cell lines, respectively.

Therefore, these findings call for the advancement of the sulfated complexes by the introduction of aromatic benzothiols to the [Re(CO)_3_]^+^ complexes stabilised by (N, S, and O) electron donors to enhance their activities, **30**–**33** [49,50].

Vitale, (2019) [51] reported potent anticancer activities of such complexes against MCF and PC3 cancer cell lines, with complexes **32** and **33** showing the highest activities with their corresponding IC_50_ values of 15.9 µM and 32.1 µM against the two cancer cell lines, respectively. On the other hand, complex **30** demonstrated activity less than 50 µM [51].

The anticancer activities of **33**–**35** were determined against A549R, HeLa, MCF, and HLF cancer cell lines (Table 6, Table 7 and Scheme 1), wherein interesting anticancer activities against cisplatin-resistant lung carcinoma (A549R) were reported [38,51].

Complex **35** (Scheme 1) showed the highest anticancer activity with a corresponding IC_50_ value of 2.1 µM against the selected cancer cell lines, on the other hand, an in vivo study of the complex in nude mice bearing A549 tumour xenografts showed a 60% reduction of the ovarian volume of the tumour tested in 21 days with the corresponding activity of 5 mgKg^−1^ [28,29].

Complex **36** revealed promising anticancer activities in vitro against a range of human cancer cell lines (Table 6). On the other hand, complex **37** had intact aqua-chloride stability, indicating its stability in in vivo studies. The complex showed a recommendable IC_50_ value of 2.2 µM and 3.0 µM against the A2780 (wild-type) and A2780CP70 (cisplatin-resistant ovarian carcinoma), respectively.

In a study carried out by Mkhatshwa et al. (2021) [6], complexes **37**–**41** showed distinct anticancer activities against a series of human cancer cell lines, i.e., HT-29, HeLa, HepG2, PT-45, A2780, and CP70 (Table 6, Table 7 and Scheme 1). The comparative study of their anticancer activities with cisplatin (**42**) showed a very close correlation, with complexes scoring higher than the positive control. Complex **39** showed the highest anticancer activity against A2780 (Ovarian epithelial carcinoma cell lines) with an IC50 value of 2.2 ± 0.2 µM in phosphate-buffered saline solvent systems. Similar activity was noticed in complex **38** with an IC_50_ value of 2.2 ± 0.2 µM in the same solvent mixtures.

Table 6 shows a comparative anticancer activity of **34**–**41** with cisplatin against a series of human cancer cell lines.

In totality varied [Re(CO)_3_]^+^ complexes with different ligands donors under different solvent systems have been used as target-specific chemotherapeutic activities for different cancer cell lines i.e., HT-29, HeLa, HepG2, PT-45, A2780, and CP70, this is so because [Re(CO)_3_]^+^ complexes coordinated with bipyridinium serves as potent chemo-theranostics owing to their activities to easily impair cells division (mitosis)[25,26,28,30,38].

Complex **43** showed positive anticancer activities against breast carcinoma, with a high drug-binding affinity of 6.7 kcal/mol, a prospective view for drug development [52]. Complex **44**, as reported by Konkankit et al. (2019) [48], showed greater activity against ovarian carcinoma with equivalent drug uptakes in nuclei, mitochondria, and the whole cancer cell, an indication that there is no drug resistance in the cancer cell lines. It was also identified that the pH plays a critical role in the drug uptake of complex **44**, with intensified luminescence at lower pH levels [48].

To enhance the activity of rhenium complexes, binuclear [Re(CO)_3_]^+^ complexes have been evaluated to determine their advancing activities against varied cancer cell lines, as well as reducing the drug resistance of the cancer cells by increasing reactive sites. Wang et al. (2019) [31] synthesised complex **45** and evaluated anticancer activities against HT-29, HeLa, HepG2, PT-45, A2780, and CP70, wherein an advanced anticancer activity with its action on mitochondria, causing oxidative stress and disruption of glutathione biosynthetic pathways and inhibiting its spread was reported (Table 6). Conversely, Pan et al. (2020) [32] evaluated the anticancer activities of two Re(I) binuclear tricarbonyl complexes of carboline derivatives, **46** and **47**, where activities of about 16-fold higher than that of cisplatin were reported. Complexes **46** and **47** work by triggering the production of reactive oxygen from the cancer cells and inducing instantaneous cell apoptosis. Irradiating **46** and **47** at 425 nm enhances their toxicity against lung carcinoma [32].

Complex **48** is generally referred to as TRIP (Tricarbonyl Rhenium Isonitrile Polypyridyl), wherein its anticancer activities against ovarian carcinoma were examined in vivo, and the studies showed that the mice treated with complex **48** in cancer xerograph had a 150% prolonged lifetime as compared to the normal tissues, an indication that the complex has a potential of prolonging the lifespan of a cancer patient by almost a double-digit hence can be a potent candidate in the development of anticancer drugs [32].

Several anticancer activities of [Re(CO)_3_]^+^ complexes have mainly been evaluated between 2019 and 2024. Several tests have been performed against varied cancer cell lines; however, there is no effective agent that has been found to act against them. The underlying toxicity towards the Vero cells (normal cells) of some of the complexes with bipyridinium moiety has adversely affected their selectivity, hence a major drawback in drug development. Cisplatin, the currently used metal-based anticancer, has been reported to have significant side effects, which may include nausea, vomiting, and hearing loss. Resistance to the drug has developed over time, a clear indication that a safer agent is needed. Mononuclear rhenium complexes have been largely tested for their anticancer activities as opposed to binuclear [Re(CO)_3_]^+^ complexes, despite binuclear species presenting more reactive sites palpable of arresting varied cancer cells; therefore, this opens another horizon to investigate the potentiality of functionalising binuclear complexes of rhenium to creating more reactive species with improved biomedical activities. Consequently, the adoption of metal–organic frameworks (MOFs) in the fight against cancer has created another avenue to unlocking the potential of organic molecules encapsulated in metal complexes, owing to their comparative advantage of being specific in action as well as their ability in drug delivery and imaging. MOFs are bringing a light of hope to developing viable anticancer agents; however, less has been done to evaluate their anticancer activities against varied cancer cell lines. In totality, the fight against cancer has proven to be an unending engagement which calls for a better approach every day, a situation that warrants an extensive look into many options for developing viable anticancer drugs.

## 3. Rhenium Labelling

While radiopharmaceuticals containing rhenium-188 or rhenium-186 radioisotopes have been extensively studied in the literature, the labelling of rhenium complexes with alternative isotopes has received far less attention. Rhenium complexes can be used to image melanoma tumours by combining them with α-MSH analogues conjugated with radiometals and radiohalogens. This improves fluorine-18 incorporation into ligands that were previously impossible to synthesise, and allows for the investigation of the mechanisms and coordination sites of rhenium through deuterium incorporation. Radiopharmaceuticals with a rhenium metal centre have been used for the treatment of breast tumours, restenosis, and atherosclerotic coronary artery disease [53,54,55,56,57,58,59], and have also found use in radio-immunotherapies treating B-cell chronic lymphocytic leukaemia, pulmonary tumours, and intensifying monoclonal antibody therapy conditioning regimens for patients about to undergo stem cell transplantation in the treatment of acute myeloid leukaemia and myelodysplastic syndrome [60,61,62,63,64]. Rhenium-labelled hydroxyethylidene-1,1-diphosphate (HEDP), in particular, is routinely used to treat painful skeletal metastases caused by breast, prostate, myeloma, and lung cancers [65].

The labelling of rhenium complexes has found widespread application in many fields, providing significant benefits in addition to the SPECT imaging and radiotherapy afforded by the rhenium-188 and rhenium-186 isotopes. To diagnose melanoma tumours using PET and SPECT imaging, highly targeted agents were radiolabelled using αMSH cyclisation with a rhenium centre. Radiolabelling studies facilitated optimisation of the molecular structures of α-MSH analogues for tumour uptake, such as substituting lysine for arginine in the peptide sequence and replacing the conjugate lysine D-enantiomer. The use of rhenium has revealed the potential to improve fluorine-18 incorporation, and labelled complexes may have new applications in PET-optical imaging. Finally, the deuteration of rhenium complexes was cleverly used to assess the diastereoselectivity of rhenium alkyloxy ligands afforded by reactions between rhenium aldehyde-coordinated complexes, to determine the atoms of cycloalkyl ligands involved in coordination to a rhenium centre, and to determine the coupling constants of rhenium deuteride-bridged complexes arising from the deuterium quadrupolar moment. Overall, the collection of these cases is a valuable resource for researchers developing new rhenium complexes for biomedical and chemical applications.

## 4. Conclusions

Re(I) tricarbonyl complexes are the new insight in the field of nanomedicine for the therapeutic and diagnostic approaches. In vitro, analysis of such complexes has been evaluated with HeLa and MCF cancer cell lines, but a few have made it through to in vivo and clinical trials, respectively. Anticancer activities of Re(I) tricarbonyl complexes have been frequently reported, with a few of which investigated for their antimicrobial activities. The majority of anti-proliferative (Re) organometallic complexes function as “traditional” chemotherapeutic agents, though a few photoactive Re complexes have recently been identified. An even larger number of (Re) organometallic compounds with PDT activity could be determined by screening the singlet oxygen production of known Re photocatalysts. Most of the complexes presented in this review are potent anticancer agents, with IC_50_ values that are equal to or greater than those of the standard cancer drug. However, the underlying toxicity and mechanisms are not always fully understood, and to our knowledge, only one in vivo study on cold Re organometallic complexes has been conducted. Undoubtedly, more extensive biological studies, both in vitro and in vivo, would provide a better understanding of the cytotoxicity of Re organometallic complexes. The hope of depleting the multidrug resistance in microbes calls for a better approach to developing potent metal complexes that may aid in antimicrobial management. The review herein gives demonstrative examples of the medical application of Re(I) tricarbonyl complexes in the treatment and management of different types of antibacterial, antifungal, antimalarial, antichagasic and anticancer activities evaluated in the last six (6) years.

Re(I) tricarbonyl complexes have shown a promising breakthrough in various medical applications and clinical trials, especially in cancer treatment, owing to their distinct properties such as photodynamic therapy and radiolabelling capacities. The commercialisation of these complexes is gaining insightful thoughts from pharmaceutical companies, researchers, and investors owing to their potential in target therapies and imaging techniques. However, their limited biodistributions in pathogenic membranes, their toxicity when used, their difficulty in synthesis, and the unpredictable stability of the metal-ion moieties in some reaction environments have all hampered their medical applications. Although the addition of rhenium binuclear complexes might improve their activity, their intricate structures prevent them from penetrating biological targets, hence limiting their bioactivities. To improve their use in drug development, a more comprehensive multidisciplinary approach is needed to address their drawbacks.

## Figures and Tables

**Table 1:** The antibacterial activities of [Re(CO)_3_(*N*,*N’*)X]^+^ against selected human pathogens.

Complexes	Bacterial Strains (MIC µgmL^−1^)		Outcomes	Reference
	*S. aureus*	*E. coli*		
**1**	50	300	The complex showed a candidate compound for the formulation of antibiotics	[8]
**2**	>300	>300	Low activity was noticed against the selected pathogens	[8]
**3**	50	50	Palpable antibacterial activity against selected test organisms was noticed. A clear indication that the complexes can be used in the formulation of antibiotics	[8]

**Table 2:** The minimum inhibitory concentration of complexes **4**–**8** against selected bacterial strains.

Complexes	MIC Values (mM)			Outcomes	References
	*S. aureus*	*E. faecium*	*E. coli*		
**4**	0.8	3.1		Potent activities against both S. aureus and E. faecium were reported with the lowest millimolar concentration of 0.8 mM. A significant anti-bacterial activity against S. aureus is noticed, with a corresponding MIC value of 0.8 mM, which is statistically comparable with the known antibiotics, linezolid, and vancomycin.	[8,9,11]
**5**	0.8	3.1		The complex had activities nearly the same as complex **4**; this could be due to their structural similarities, with only variation on the side chain lactone ring. Its activities were also comparable to those of the positive control, having promising antibacterial applications.	[8,9,11]
**6**	0.8	3.1		A probable antibacterial agent with promising activities both in vitro and in vivo.	[8,9,11]
**7**	0.8	3.1		The introduction of more electron donors, namely. Oxygen and nitrogen, in the complex, help to arrest and inhibit microbial colonies within the system. This complex has several active sites, and hence reduces multidrug resistance.	[8,9,11]
**8**	0.8	6.1		It is reported that this complex exhibited activities that are not different from the positive control; consequently, in vivo tests also affirm this in zebrafish tests.	[8,9,11]
**9**	0.002	-	0.023	The complex showed activity twice as compared as the positive control. It also had no variation in its activities when MRSA and colistin-resistant *E. coli* strains were used. The toxicological reports affirm that the complex is safe and shows no hemolysis even up to a concentration of 300 µM, hence it can be used as a prospective antibiotic.	[8,9,11]
**10**	0.01	-	>64	The reduced form of complex **9** showed a reduction in activity; it exhibited very low activity against the Gram-negative *E. coli,* but showed better activity against the Gram-positive bacterium strain *S. aureus*.	[8,9,11]
**11**	0.002	-	>64	The hydrogenated form of complex **10**, a derivative of bis-quinoline, showed a noticeable activity against *S. aureus* at micromolar concentrations; however, poor activity was reported against the Gram-negative bacterium strain of *E. coli.*	[8,9,11,12]
Linezolid	0.7	-		-	[8,9,11,13,14]
Vancomycin	0.7	-		-	[8,9,11,13,14]
Polymyxin	0.0005	-	0.0002	-	[8,9,11,13,14]

**Table 3:** MIC (mg/mL) of complexes (**4**–**8, 12** and **13**) against *Candida albicans*, *Candida krusei*, *Candida glabrata*, *Candida parapsilosis*, and *Cryptococcus neoformans*.

Complexes	MIC (mg/mL)					References
	*C. albicans*	*C. glabrata*	*C. krusei*	*C. parapsilosis*	*C. neoformans*	
**4**	22.50	22.50	11.20	22.50	-	[9,16,17]
**5**	11.20	22.50	4.50	11.20	-	[9,16,17]
**6**	13.50	13.50	13.50	<7.00	-	[9,16,17]
**7**	12.90	12.90	12.90	12.90	-	[9,16,17]
**8**	4.90	4.90	4.90	4.90	-	[9,16,17]
**12**	0.00062	-	-	-	-	[9,16,17]
**13**	0.032	-	-	-	0.032	[9,16,17]

**Table 4:** IC_50_ values of complexes (**14**–**16**) against *P. falciparum* and a resistant isolate of *P. falciparum* (L1) as compared to the positive control chloroquine diphosphate (CDP).

Complexes	IC_50_ Value (µM)		
	*P. falciparum*	L1	References
14	4.61	-	[18,19]
15	0.356	0.611	[18,19]
16	0.441	1.80	[18,19]
CDP	0.014	0.247	[18,19]

**Table 5:** Antichagasic activities of complexes **16** to **19** against *T. cruzi epimastigotes* ± SD (μM) and their corresponding selectivity indexes.

Complex	Antichagasic Activities IC_50_ T. Cruzi Epimastigotes ± SD (μM)	Selectivity Index Epimastigotes	References
**16**	9.42 ± 1.53	0.34	[20,21,22]
**17**	8.43± 2.20	1.5	[20,21,22]
**18**	3.48 ± 0.98	0.89	[20,21,22]
**19**	8.48 ± 1.46	1.6	[20,21,22]

**Table 6:** A summarised anticancer activity of different Re(I) tricarbonyl complexes (**34**–**42**), IC_50_ in µM.

Cell Line	34	35	36	37	38	39	40	41	42	References
KB-3-1	–	–	0.92 ± 0.2	–						[28,31,32,33]
KBCP20	–	–	1.6 ± 0.4	–						[31,32,33]
A2780	–	–	2.2 ± 0.2	3.5 ± 2.8	2.2 ± 0.8	2.2 ± 0.2	–	–	0.23 ± 0.07	[31,32,33]
A2780 CP70	–	–	3.0 ± 0.7	–			–	–	–	[31,32,33]
A549	133.2 ± 4.3	2.2 ± 0.2	6.7 ± 4.9	–	–	–	–	–	–	[31,32,33]
AF49 CisR	–	2.1 ± 0.1	5.4 ± 1.8	–	–	–	–	–	–	[31,32,33]
H460	–	–	4.5 ± 0.7	–	–	–	–	–	–	[31,32,33]
H460 CisR	–	–	5.3 ± 2.9	–	–	–	–	–	–	[31,32,33]
MRC-5	–	–	4.1 ± 0.9	–	–	–	–	–	–	[31,32,33]
HeLa	126.4 ± 2.8	1.8 ± 0.2	1.2 ± 0.2	–	–	–	–	–	6.6 ± 0.7	[31,32,33]
MCF-7	51.4 ± 3.0	2.2 ± 0.2	–	–	–	–	–	–	–	[31,32,33]
T98G				–	–	–	–	>50		[31,32,33]
PC3	59.4 ± 3.8	–	–	–	–	–	–	>50	2.19 ± 0.11	[31,32,33]
HepG2	–	–	–	–	–	–	–	–	10.5 ± 0.5	[31,32,33]
LO_2_	–	–	–	–	–	–	–	–	–	[31,32,33]
HLF	–	12.7 ± 0.8	–	–	–	–	–	–	–	[31,32,33]
MDA-MB-231	48.5 ± 2.8	–	–	–	–	–	–	–	–	[31,32,33]
PT-45				–	–	–	>250		2.2 ± 0.3	[31,32,33]
HT-29	47.5 ± 0.9	–	–	–	–	–	>250		32.6 ± 0.7	[31,32,33]

**Table 7:** Cancer Cell Line Description.

Cell Line	Description
HeLa	Cervical cancer cell
A2780	Human ovary epithelial cell, ovarian endometrioid adenocarcinoma
HT-29	Human colon epithelial cell, adenocarcinoma.
PT-45	Human pancreas epithelial cell, adenocarcinoma.
T98G	Human brain fibroblast, glioblastoma.
PC3	Human prostate epithelial cell, adenocarcinoma
HepG2	Human liver epithelial cell, hepatocellular carcinoma.
KBCP20	Breast cancer carcinoma
A2780CP70	Ovarian endometrioid adenocarcinoma
A549	Lung carcinoma epithelial cells
H460	Type II pulmonary epithelium carcinoma
H460CisR	Lewis lung carcinoma
MRC-5	Fetal lung fibroblast cell carcinoma
MCF-7	Breast cancer
LO2	Human fetal hepatocyte cell line
HLF	Liver carcinoma cell lines
MDA-MB-231	Epithelial, human breast cancer cell line
